# Medical Male Circumcision Is Associated With Improvements in Pain During Intercourse and Sexual Satisfaction in Kenya

**DOI:** 10.1016/j.jsxm.2017.02.014

**Published:** 2017-04

**Authors:** Monica P.C. Nordstrom, Nelli Westercamp, Walter Jaoko, Timothy Okeyo, Robert C. Bailey

**Affiliations:** 1 Division of Epidemiology and Biostatistics, University of Illinois at Chicago, Chicago, IL, USA; 2 Department of Medical Microbiology, University of Nairobi, Nairobi, Kenya; 3 Nyanza Reproductive Health Society, Kisumu, Kenya

**Keywords:** Male Circumcision, HIV Infection, Sexual Dysfunction, Orgasm, Erectile Dysfunction, Premature Ejaculation

## Abstract

**Background:**

Two cohort studies using data from randomized controlled trials in Africa offer the best evidence to date on the effects of voluntary medical male circumcision (VMMC) on male sexual function and satisfaction, suggesting no significant impairments in sexual function or satisfaction and some improvements in sexual function after male circumcision.

**Aim:**

To assess the effects of VMMC on sexual function and satisfaction in a large population-based cohort of men circumcised as adults and uncircumcised controls in Kenya.

**Methods:**

Sexual function and satisfaction of young (median age = 20 years) sexually active men (1,509 newly circumcised men and 1,524 age-matched uncircumcised controls after 5% loss to follow-up) were assessed at baseline and 6, 12, 18, and 24 months, with data collected in 2008 to 2012. Self-reported data on lack of sexual interest or pleasure, difficulty getting or maintaining erections, orgasm difficulties, premature ejaculation, pain during intercourse, and satisfaction with sexual intercourse were analyzed with mixed-effect models to detect differences between circumcised and uncircumcised men and changes over time.

**Outcomes:**

Changes over time in sexual interest, desire and pleasure, erectile and ejaculatory function, and pain during intercourse (dyspareunia) in circumcised and uncircumcised men; group differences in time trends; satisfaction with sexual performance; and enjoyment of sex before and after circumcision.

**Results:**

Sexual dysfunctions decreased in the two study groups from 17% to 54% at baseline to 11% to 44% at 24 months (*P* < .001), except dyspareunia, which decreased only in circumcised men (*P* < .001). Sexual satisfaction outcomes increased in the two study groups from 34% to 82% at baseline to 66% to 93% at 24 months (*P* < .001), with greater improvements in circumcised men (*P* < .001). On average, 97% of circumcised men were satisfied with sexual intercourse and 92% rated sex as more enjoyable or no different after circumcision compared with before circumcision.

**Clinical Translation:**

Results are applicable to VMMC programs seeking to increase the acceptability of male circumcision as part of comprehensive HIV prevention.

**Strengths and Limitations:**

Large-scale population-based longitudinal data restricted to sexually active individuals and adjusted for differences in baseline levels of outcomes and potential confounders are used. The questionnaire used, although not a standardized survey instrument, includes all major domains of male sexual function and satisfaction used in the most common standardized tools.

**Conclusions:**

Results are consistent with large cohort studies of VMMC using data from randomized controlled trials and indicate that VMMC has no significant detrimental effect or might have beneficial effects on male sexual function and satisfaction for the great majority of men circumcised as adults.

**Nordstrom MPC, Westercamp N, Jaoko W, et al. Medical Male Circumcision Is Associated With Improvements in Pain During Intercourse and Sexual Satisfaction in Kenya. J Sex Med 2017;14:601–612.**

## Introduction

Continued scale-up of voluntary medical male circumcision (VMMC) is needed to decrease HIV transmission in 14 priority African countries and will depend in part on the continued acceptability of VMMC programs to target populations. The possibility that VMMC has deleterious effects on sexual function and satisfaction could affect the future acceptability and uptake of VMMC programs.^1^, ^2^, ^3^, ^4^

Various biological mechanisms have been proposed that could lead to losses in penile sensitivity or sensation after circumcision. These include a smaller skin surface, neural reorganization and/or atrophy of penile nerves,^5^ keratinization of the skin,^6^, ^7^, ^8^, ^9^ loss of the natural gliding mechanism and lubrication provided by the foreskin, and loss of smegma with resulting loss of pheromones.^10^ Physiologic changes, if present, have been interpreted as potentially negative, as in possibly decreasing sexual pleasure,^9^ or potentially positive, as in increasing intravaginal ejaculatory latency time and resulting in better ejaculation control and prolonged sexual pleasure.^11^ These contrary views have led to controversy over the effects of VMMC on male sexual function and satisfaction.

Two cohort studies using data from randomized controlled trials (RCTs) in Africa^12^, ^13^ offer the best evidence to date on the effects of VMMC on male sexual function and satisfaction, suggesting no significant impairments in sexual function or satisfaction and some improvements in sexual function after male circumcision. However, RCT conditions can differ from VMMC program conditions in ways that could affect men's self-assessment of sexual function and satisfaction, such as randomization of participants into study groups, differences in perceptions about VMMC before and after RCT results are available, and differences in staff training, medical equipment, and implementation procedures.^14^, ^15^ The purpose of this study was to add evidence garnered in the setting of a national program scaling up VMMC for HIV prevention. We provide analyses of large-scale population-based longitudinal data on the sexual function and satisfaction of men circumcised as adults, including data from before and after circumcision.

## Methods

### Recruitment of Research Participants and Data Collection

Data for this study were collected by Westercamp et al^15^ during initial VMMC program implementation in Kenya. Participants were uncircumcised young men 18 to 35 years old who were recruited in 2008 to 2010, resided in Kisumu, Kenya and surrounding districts, and had no plans to relocate in the following 2 years. Study information was posted at VMMC facilities and distributed by word of mouth and through community outreach. Men seeking circumcision services at VMMC facilities were enrolled in the intervention group, which required circumcision through the Kenyan VMMC program for participation. Men who did not wish to be circumcised were encouraged to enroll as controls and were matched by frequency to VMMC participants in age and community and of residence. Participants were not included or excluded based on pre-existing medical conditions, HIV status, or levels of sexual activity; however, participants with obvious indications for circumcision were excluded from the study and referred for therapeutic circumcision.

All participants provided signed informed consent and completed a survey questionnaire at baseline. Follow-up visits were conducted at nine health facilities providing VMMC services or at participants' homes, workplaces, or other convenient location at 6, 12, 18, and 24 months after enrollment within a period of ±3 months. Participants who missed a study visit (>3 months late) were allowed to continue participation in the study. At each visit, participants were visually examined to confirm circumcision status, asked to complete a study questionnaire, and paid approximately $2.50 compensation for missed worktime and transportation. Questionnaires were administered using audio computer-assisted self-interview (70%) or a paper form (30%).

The behavioral questionnaire^15^ incorporated the major domains of sexual function—sexual drive and desire, erectile function, ejaculation, orgasm, and sexual satisfaction—covered by the three validated survey instruments most commonly used to diagnose male sexual dysfunctions—the Brief Male Sexual Function Inventory, the International Index of Erectile Function, and the Premature Ejaculation Diagnostic Tool^16^, ^17^, ^18^, ^19^, ^20^—and included questions referring to pain during intercourse (dyspareunia). The assessment of sexual function and satisfaction matched the questions asked in the Kisumu, Kenya RCT of male circumcision for direct comparability. Survey questions referred to symptoms persisting over a period of at least 2 weeks in the past 6 months. Sexual function questions asked participants whether they had experienced lack of sexual interest or pleasure, difficulty getting or maintaining erections, not being able to achieve an orgasm, achieving an orgasm too quickly, and pain during intercourse. Sexual satisfaction questions asked participants how satisfied they were with sexual intercourse, level of sexual desire, getting erections, maintaining erections, interval between erections, ease in ejaculation, and level of pain during intercourse using a comparable five-item satisfaction response scale ranging from “very dissatisfied” to “very satisfied.”

No direct risk reduction counseling was given by study staff at study visits. However, participants in the two study groups were encouraged to use the HIV testing and counseling services and were exposed to educational videos on HIV in health facility waiting areas. In addition, conforming to Kenyan national guidelines for provision of VMMC,^21^ circumcised men received risk reduction and partial protection counseling at the time of the procedure.

### Sample Sizes and Power

Participants who had only baseline data (approximately 5% of recruited participants) were excluded from longitudinal analyses. Analyses were restricted in the two study groups to sexually active participants, defined as those who answered “yes” to having had intercourse in the past 6 months, so that participants who reported becoming sexually active at any time during the study were included at each successive visit. At baseline, all participants were uncircumcised and study group refers to group assignment. At follow-up visits, study group refers to actual circumcision status.

For cross-sectional comparisons of two proportions, we estimated at least 80% power to detect effect sizes of at least 0.20. For longitudinal comparisons of two proportions, we estimated at least 80% power to detect effect sizes of at least 0.20 with repeated measures correlations of at least 0.5, assuming five follow-up visits, random linear trends with AR1 variance-covariance structure, 21% crossovers (unequal group sizes), two-tailed tests, and an α value equal to 0.05. RMASS2 software was used for calculations.^22^

### Data Analysis

All outcomes of sexual function and satisfaction were dichotomized into binary responses (“yes” or “no” and “satisfied” or “dissatisfied”) to simplify interpretation of results. Fewer than 2.5% of data were missing for any outcome variable. “Don't know,” “refused to answer,” and “not applicable” responses were grouped with missing responses. Random-intercept mixed models were used to adjust for subject differences in outcomes at baseline. Time effect at each follow-up visit was estimated using the baseline visit as reference. Group effects were estimated for the VMMC group using the control group as reference.

Demographic characteristics and sexual behaviors were assessed for crude baseline associations with the exposure and outcomes using Wilcoxon Mann-Whitney test for continuous, non-normally distributed data and Pearson χ^2^ test for categorical data at significance level of an α value equal to 0.10. To increase comparability between models, all multivariable models were adjusted for potential demographic confounders.

Sensitivity analysis was conducted to assess whether significant differences in main effects occurred when including vs excluding crossover participants (21% control to VMMC, 8% VMMC to control). Excluding crossover participants in models yielded no significant changes in the relevant main effects of time, group, and time-by-group interaction. Therefore, results for as-treated analysis, including all participants, are presented.

SAS 9.4 was used for baseline comparisons and longitudinal analysis (PROC NLMIXED logistic regression).^23^ The study was approved by the ethics and research committee of the Kenyatta National Hospital (Kenyatta, Kenya) and the institutional review board of the University of Illinois (Chicago, IL, USA).

## Results

### Baseline Characteristics of Study Participants

Compared with participants who returned for at least one follow-up visit, participants lost to follow-up (approximately 5% in each group) had significantly lower prevalence of secondary education (65% vs 73%; *P* = .04), Luo ethnicity (96% vs 98%; *P* = .03), and sexual activity in the past 6 months (57% vs 67%; *P* = .02).

Demographic characteristics and sexual behaviors of study participants and their baseline crude associations with the group exposure are presented in Table 1. Men choosing to be circumcised were slightly younger and were more likely to have at least some secondary education, to be unemployed, to be unmarried, and to identify as having non-Luo ethnicity.

Because previous studies showed that men can vary in their assessment of how problematic a symptom is,^17^ we assessed sexual dysfunctions and sexual satisfaction separately. Sexual function and satisfaction outcomes and their baseline crude associations with group exposure are presented in Table 2. Sexual dysfunctions ranged from 17% for pain during intercourse in the two groups to 52% and 54% for premature ejaculation in the VMMC and control groups, respectively. There were no significant differences between groups in baseline prevalence of sexual dysfunctions, which were relatively high and comparable to those found in most large studies on the topic. Sexual satisfaction ranged from 34% and 36% for level of pain during intercourse to 77% and 80% for getting erections in the VMMC and control groups, respectively. The VMMC group had lower rates of satisfaction with sexual intercourse, level of sexual desire, interval between erections, and ease in ejaculation.

### Changes in Sexual Function and Satisfaction in Circumcised and Uncircumcised Men

The estimates of effect for each variable for participants with the same baseline values of the outcome, by group, are listed in Tables 3 and 4.

**Figure 1:**
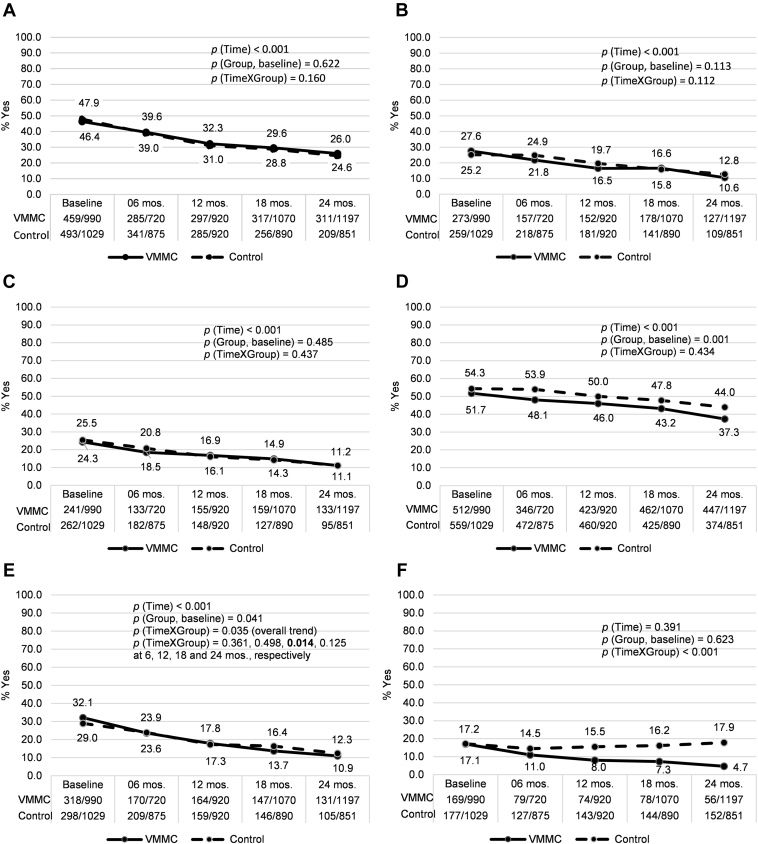
Proportions of reported sexual function outcomes among sexually active men, by group and follow-up visit. *P* values for the effects of time (overall trend), group (VMMC at baseline, compared to control group) and time-by-group interaction are based on unadjusted analyses. For lack of pleasure during sex (E), despite the significant overall time trend, groups only differed significantly at the 18-month follow-up visit. VMMC = voluntary medical male circumcision.

After adjusting for demographic variables and condom use, there were no significant group differences in prevalence of sexual dysfunctions at baseline, except for premature ejaculation, which was slightly more prevalent in the control group (54% vs 52%; *P* = .001). Measurements of sexual dysfunction decreased over time in the two groups starting at 6 months and continuing through 24 months of follow-up, except for pain during intercourse, which decreased only in the VMMC group starting at 6 months (*P* < .001; Table 3, Figure 1).

**Figure 2:**
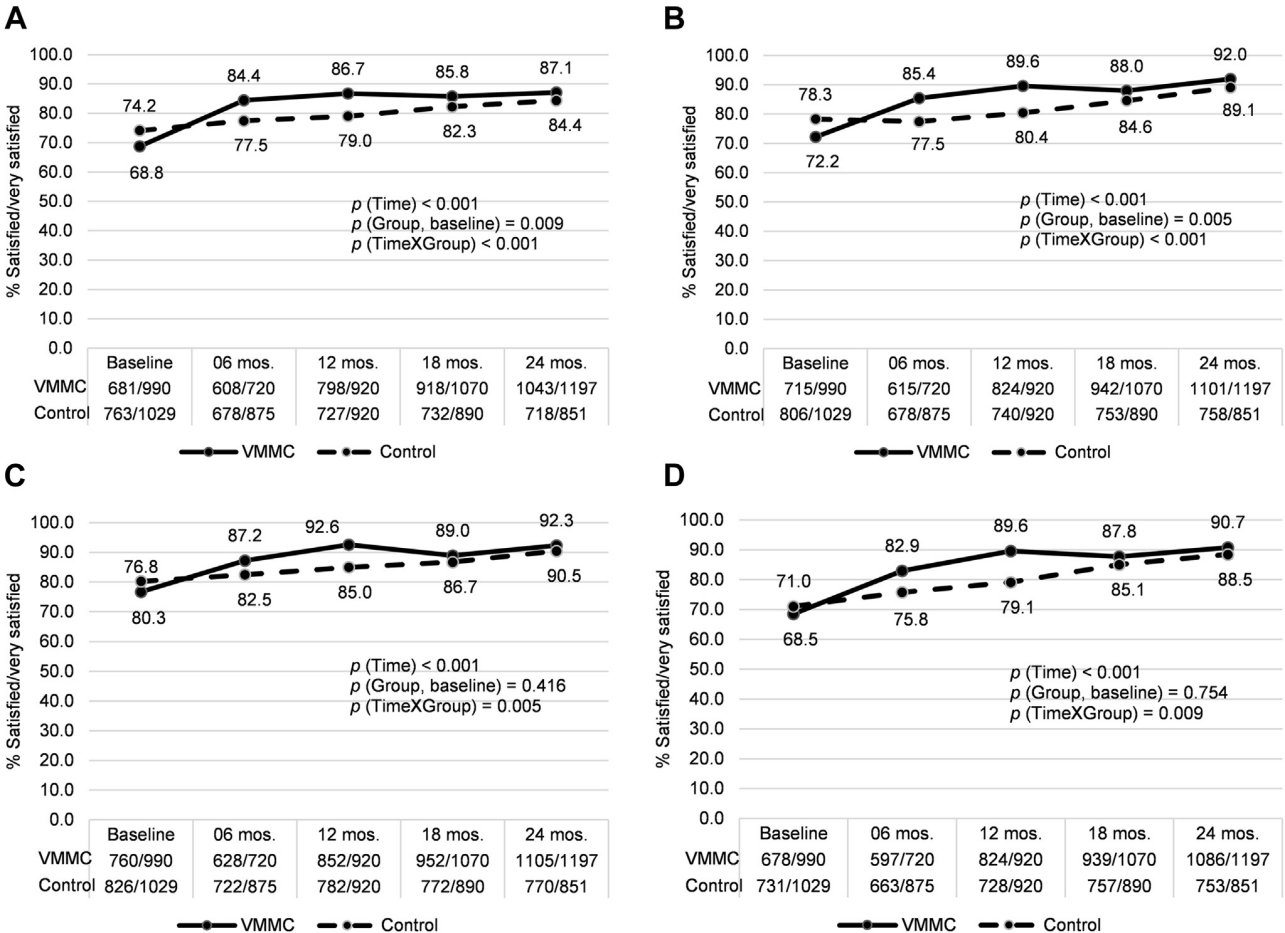

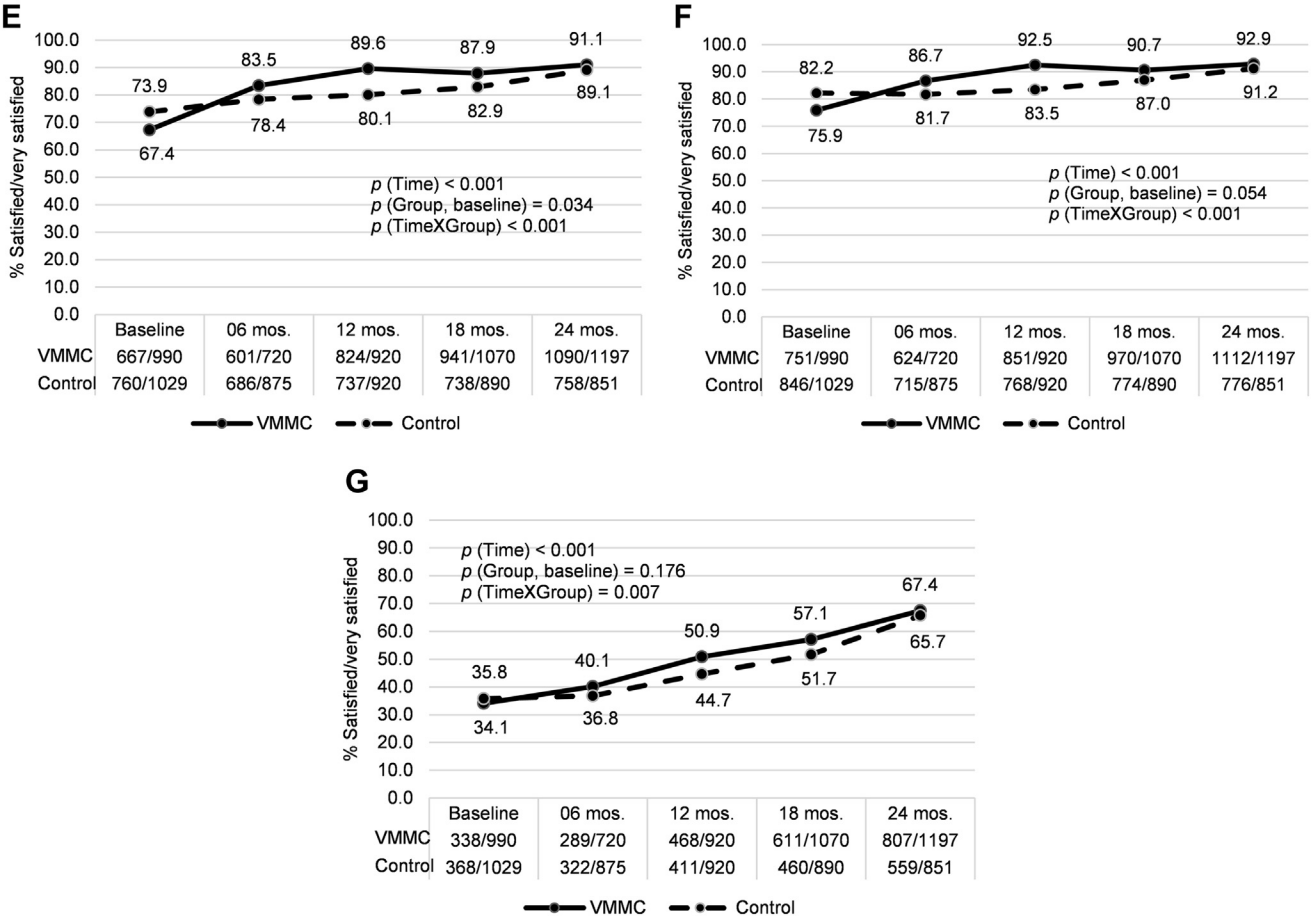
Proportions of reported sexual satisfaction outcomes among sexually active men, by group and follow-up visit: (A) Satisfaction with sexual intercourse, (B) Satisfaction with level of sexual desire, (C) Satisfaction getting erections, (D) Satisfaction maintaining erections, (E) Satisfaction with interval between erections, (F) Satisfaction with ease of ejaculation and (G) Satisfaction with level of pain during intercourse. *P* values for the effects of time (overall trend), group (VMMC at baseline, compared to control group) and time-by-group interaction are based on unadjusted analyses. VMMC = voluntary medical male circumcision.

At baseline, after adjusting for demographic variables, the VMMC group had significantly lower prevalence of satisfaction with sexual intercourse (*P* = .009), level of desire (*P* = .005), interval between erections (*P* = .034), and ease of ejaculation (*P* = .054). All outcomes of sexual satisfaction improved over time in the two study groups, with significantly greater improvements in the VMMC group starting at 6 months and continuing through 24 months of follow-up (Table 4, Figure 2).

### Sexual Satisfaction After Circumcision

A four-visit average of 97% of participants reported being satisfied or very satisfied with sexual performance after circumcision, 92% reported that sex was better or no different after circumcision, and no more than 2% had no opinion or missing responses. These high levels of sexual satisfaction persisted, tended to increase through the 24 months of follow-up, and were consistent with the significantly greater improvements in sexual satisfaction outcomes in circumcised men compared with uncircumcised controls.

## Discussion

Most studies comparing sexual function and satisfaction in circumcised and uncircumcised men have used self-reported cross-sectional survey data. Some large nationally representative cross-sectional surveys^24^, ^25^ have included countries with high and low male circumcision rates, controlling for demographic factors that might influence attitudes toward circumcision. Several studies have compared intravaginal ejaculatory latency time^11^, ^24^, ^25^, ^26^, ^27^, ^28^ and a few small studies have reported direct physiologic measurements of penile sensitivity and sexual response^27^, ^29^, ^30^, ^31^ in circumcised and uncircumcised men, finding no evidence for detrimental physiologic effects of male circumcision. Two cohort studies using data from RCTs^12^, ^13^ in Africa offer the best evidence to date on the effects of VMMC on male sexual function and satisfaction, suggesting no significant impairments in sexual function or satisfaction and some improvements in sexual function after male circumcision. The most recent and comprehensive systematic review thus far, published in 2013 by Morris and Krieger,^32^ found no evidence for differences between circumcised (n = 20, 931) and uncircumcised (n = 19, 542) men in any component of sexual function, satisfaction, or sensitivity and pleasure. The cumulative evidence from cross-sectional, case-control, and pre-post circumcision studies suggests that medical circumcision might have little or no effect on male sexual function and satisfaction, but there is need for additional, large-scale, population-based evidence in the context of a national program to scale-up VMMC for HIV prevention.

We found that VMMC was associated with improvements in pain during intercourse and sexual satisfaction and was not associated with any other sexual dysfunction over 24 months of follow-up. These results are consistent with the large cohort studies of VMMC conducted in African countries. The study by Krieger et al^13^ using data from the Kenyan RCT is the most comparable to ours in terms of the population surveyed and outcomes of sexual function assessed, which included premature ejaculation, erectile dysfunction, dyspareunia, inability to ejaculate, lack of pleasure during sex, normal feeling of erections, deviation during erection, and difficulty achieving erection because the skin was too tight. They found no association between circumcision and any sexual dysfunction or self-reported decreases in penile sensitivity. As in the present study, Krieger et al^13^ found significant decreases in reported sexual dysfunctions at the 24-month study visit compared with baseline in the circumcised and control groups (circumcised, from 23.6% to 6.2%; uncircumcised, from 25.9% to 5.8%; *P* for linear trend < .001; *P* for quadratic trend < .02), albeit with no significant group difference in rates of dyspareunia over time. In Uganda, Kigozi et al^12^ used RCT data to compare sexual function and satisfaction in circumcised men (n = 2,210) and uncircumcised controls (n = 2,246) 15 to 49 years old. They found very low rates of sexual dysfunctions and dissatisfaction in men overall (<2%), whereas rates of sexual dysfunction found in the Kenyan RCT were higher (24.7% overall at baseline, 95% CI = 23.0–26.5) and generally comparable to rates found in our study and in most other countries.^24^, ^25^, ^33^, ^34^, ^35^, ^36^, ^37^ Differences between Kenya and Uganda in cultural views about sexuality and survey tools used in each study might have contributed to the comparably low rates of sexual dysfunction and dissatisfaction reported by Kigozi et al. The proportion of men reporting being satisfied or very satisfied significantly increased in the control group (99.9% at study end), whereas no significant change over time was found in the circumcision group. As in our study, VMMC was associated with a decrease in dyspareunia (from 1.2% to 0.1%; *P* < .001). VMMC also was associated with small decreases in erectile problems (from 0.8% to 0.3%; *P* value not available) and difficulties with penetration (from 1.5% to 0.6%; *P* < .001).

The lower baseline rates of sexual satisfaction we found in the VMMC group suggest that some sexual problems not detected during enrollment or through our survey instrument might have been more prevalent in this group, contributing to some self-selection bias. VMMC programs could address this issue by providing medical screening and diagnosis of sexual dysfunctions to men in their communities, regardless of intent to be circumcised, and referring men for therapeutic circumcision when appropriate.

Improvements in sexual function and satisfaction in the two study groups could be due in part to participants becoming older and more sexually experienced during the course of the study.

## Limitations

Men with more positive perceptions of male circumcision and/or lower levels of satisfaction at baseline might have been more likely to choose to be circumcised (self-selection bias) and to report improvements in satisfaction after circumcision compared with men with less positive perceptions of male circumcision and/or higher levels of sexual satisfaction (reporting bias). Even if this were the case, it is not likely that more positive views of circumcision in men choosing to be circumcised would have influenced their self-assessment of sexual function to the extent of masking detrimental effects of circumcision, had they been present, because these are relatively objective measurements of sexual experience—pain, erection, and orgasm difficulties—compared with feelings of sexual satisfaction. Although the lower baseline levels of sexual satisfaction in the VMMC group suggest some self-selection bias occurred, circumcision could have contributed to improvements in satisfaction in circumcised men by decreasing rates of dyspareunia. No physiologic measurements were used; however, the self-reported outcome measurements used in this study are widely accepted and comparable to those used in other large studies of male sexual function and satisfaction. The survey instrument used in this study was not a standardized, validated diagnostic tool, and terms such as *premature ejaculation* and *erectile dysfunction* are based on reporting of the main symptom(s) indicative of these conditions and are not expected to be equivalent to clinical diagnoses. The assessment of sexual function and satisfaction used in this study, although not formally validated in this population linguistically, was chosen to match the questionnaire used in the Kisumu RCT of male circumcision, thus allowing for a direct comparison of the findings. Although comparability between groups was maximized by adjusting all models for identified confounders and by controlling for subject effects at baseline, residual confounding might exist. Exposure to educational videos about HIV and HIV testing and counseling at VMMC facilities might have influenced perceptions about VMMC in study participants. Research participants came almost exclusively from one ethnic group and geographic region of Kenya and might not be representative of men from other regions of Kenya or of East and Southern Africa, where VMMC programs are being scaled up.

## Conclusions

Although specific outcomes vary among studies, results from this study and from other comparable prospective cohort studies conducted in African countries indicate that medical male circumcision has no significant detrimental effect or might have beneficial effects on male sexual function and satisfaction for the great majority of men circumcised as adults. These results are applicable to VMMC programs seeking to increase the acceptability of male circumcision as part of comprehensive HIV prevention. In addition, the high baseline rates of sexual dysfunctions we found in the two study groups and lower baseline rates of sexual satisfaction in men seeking circumcision services compared with controls suggest that medical screening for sexual dysfunctions and recommendation for therapeutic circumcision or other appropriate treatment also might be beneficial to men in these communities.

## Statement of authorship

**Category 1**
(a) Conception and Design
  Nelli Westercamp; Walter Jaoko; Timothy Okeyo; Robert C. Bailey
(b) Acquisition of Data
  Nelli Westercamp; Walter Jaoko; Timothy Okeyo; Robert C. Bailey
(c) Analysis and Interpretation of Data
  Monica P.C. Nordstrom; Nelli Westercamp; Robert C. Bailey

**Category 2**
(a) Drafting the Article
  Monica P.C. Nordstrom
(b) Revising It for Intellectual Content
  Nelli Westercamp; Walter Jaoko; Timothy Okeyo; Robert C. Bailey

**Category 3**
(a) Final Approval of the Completed Article
  Monica P.C. Nordstrom; Nelli Westercamp; Walter Jaoko; Timothy Okeyo; Robert C. Bailey

## Figures and Tables

**Table 1:** Demographic and sexual behavior characteristics of study participants at baseline by group

	VMMC group (n = 1,588)	Control group (n = 1,598)	*P* value
Demographic characteristics∗†			
Age (y)	20 (19–24; 18–35)	20 (19–24; 18–35)	.081¶
Age (matching intervals)			.095¶
18–24 y	1,259 (79)	1,231 (77)	
25–29 y	227 (14)	233 (15)	
30–35 y	102 (6)	134 (8)	
Religion			.999
Catholic	503 (32)	506 (32)	
Anglican, Pentecostal, or 7th Day Adventist	722 (45)	727 (45)	
Other	363 (23)	365 (23)	
Ethnic group			<.001¶
Luo	1,547 (97)	1,585 (99)	
Other	41 (3)	13 (1)	
Educational level			<.001¶
Primary or less	367 (23)	510 (32)	
Any secondary or higher	1,221 (77)	1,088 (68)	
Employment status			<.001¶
Employed	421 (27)	584 (37)	
Unemployed	1,167 (73)	1,014 (63)	
Marital status			<.001¶
Single	1,097 (69)	994 (62)	
Married or living as married	491 (31)	604 (38)	
Sexual behaviors∗†			
Age at first sexual experience (y)‡	16 (15–18; 9–30; 1,388)	16 (15–18; 9–29; 1,424)	.342
Sexual intercourse in past 6 mo§			.194
Yes	1,032 (65)	1,074 (67)	
No	555 (35)	524 (33)	
Condom use at most recent sexual encounter‖			.715
Yes	459 (48)	477 (47)	
No	500 (52)	537 (53)	

VMMC = voluntary medical male circumcision.

∗ Data are presented as median (interquartile range; range) for continuous data and number (percentage) for categorical data. *P* values (one-sided) for study group comparisons are based on Wilcoxon Mann-Whitney (Wilcoxon rank-sum) test for non-normally distributed continuous data and Pearson χ^2^ test for categorical data. All subjects were uncircumcised at baseline; study group refers to participants' choice of enrolling in the VMMC or control group.

† Total number of responses for each outcome differs slightly from the total number of participants in the study group depending on the number of sexually active participants and missing responses (missing, don't know, refuse to answer, and not applicable are grouped together).

‡ Missing data for age at first sexual experience are 200 observations (13%) and 174 observations (11%) for the VMMC and control groups, respectively.

§ Analysis of sexual function and satisfaction outcomes is restricted to sexually active subjects at any study visit, defined as subjects who reported having sexual intercourse in the past 6 months.

‖ Although not associated with group choice at baseline, condom use increased significantly over time in the two study groups (*P* < .001), with a larger increase in the VMMC group (*P* < .001). Therefore, it is associated with circumcision (exposure) over time.

¶ Crude associations with *P* values less than or equal to .10.

**Table 2:** Prevalence of sexual function and satisfaction outcomes at baseline by group

Outcome variables	VMMC group (n = 1,588)	Control group (n = 1,598)	*P* value
Sexual function outcomes∗†			
Lacked interest in sex			.357
Yes	459 (46)	493 (48)	
No	528 (53)	529 (51)	
Missing	3 (0)	7 (1)	
Was unable to come to a climax (orgasm difficulties)			.373
Yes	241 (24)	262 (25)	
No	722 (73)	748 (73)	
Missing	27 (3)	19 (2)	
Came to a climax too quickly (premature ejaculation)			.376
Yes	512 (52)	559 (54)	
No	447 (45)	445 (43)	
Missing	31 (3)	25 (2)	
Experienced pain during intercourse (dyspareunia)			.578
Yes	169 (17)	177 (17)	
No	810 (82)	835 (81)	
Missing	11 (1)	17 (2)	
Did not find sex pleasurable			.277
Yes	318 (32)	298 (29)	
No	649 (66)	709 (69)	
Missing	23 (2)	22 (2)	
Had trouble achieving or maintaining erection (erectile dysfunction)			.352
Yes	273 (28)	259 (25)	
No	701 (71)	748 (73)	
Missing	16 (2)	22 (2)	
Sexual satisfaction outcomes∗†			
Sexual intercourse			.017‡
Satisfied or very satisfied	681 (69)	763 (74)	
Dissatisfied or very dissatisfied	279 (28)	233 (23)	
Missing	30 (3)	33 (3)	
Level of sexual desire			.005‡
Satisfied or very satisfied	715 (72)	806 (78)	
Dissatisfied or very dissatisfied	240 (24)	200 (19)	
Missing	35 (4)	23 (2)	
Getting erections			.129
Satisfied or very satisfied	760 (77)	826 (80)	
Dissatisfied or very dissatisfied	198 (20)	179 (17)	
Missing	32 (3)	24 (2)	
Maintaining erections			.451
Satisfied or very satisfied	678 (68)	731 (71)	
Dissatisfied or very dissatisfied	284 (29)	270 (26)	
Missing	28 (3)	28 (3)	
Interval between erections			.005‡
Satisfied or very satisfied	667 (67)	760 (74)	
Dissatisfied or very dissatisfied	281 (28)	239 (23)	
Missing	42 (4)	30 (3)	
Ease of ejaculation			.002‡
Satisfied or very satisfied	751 (76)	846 (82)	
Dissatisfied or very dissatisfied	202 (20)	158 (15)	
Missing	37 (4)	25 (2)	
Level of pain on intercourse			.745
Satisfied or very satisfied	338 (34)	368 (36)	
Dissatisfied or very dissatisfied	571 (58)	578 (56)	
Missing	81 (8)	83 (8)	

VMMC = voluntary medical male circumcision.

∗ Data are presented as number (percentage). *P* values for study group comparisons are based on Pearson χ^2^ test. The total number of responses for each outcome differs slightly from the total number of participants in the study group depending on missing responses (missing, don't know, refuse to answer, and not applicable are grouped together).

† Longitudinal analyses of outcomes are restricted to sexually active subjects at any follow-up visit, defined as subjects who reported having sexual intercourse in the past 6 months. All subjects were uncircumcised at baseline. Study group at baseline refers to participants' choice of enrolling in the VMMC or control group.

‡ Crude associations with *P* values less than or equal to .10.

**Table 3:** Changes in sexual function in circumcised and uncircumcised men over 12 and 24 months of follow-up: results from adjusted random-intercept logistic regression models∗

Outcome and parameters	Estimate (β)	Standard error	Pr > |t|
Lack of interest in sex			
12 mo	−0.8	0.08	<0.001†‖
24 mo	−1.2	0.1	<0.001†‖
VMMC	0.0	0.1	0.622
Employment	−0.1	0.1	0.033
Erectile dysfunction			
12 mo	−0.7	0.1	<0.001†‖
24 mo	−1.3	0.1	<0.001†‖
VMMC	0.0	0.1	0.741
Age 25–29 y	0.3	0.1	0.007‖
Age 30–35 y	0.4	0.2	0.007‖
Secondary education	−0.4	0.1	<0.001‖
Marital status	0.3	0.1	<0.001‖
Condom use at most recent sexual encounter	−0.2	0.1	0.018§‖
Orgasm difficulties			
12 mo	−0.7	0.1	<0.001†‖
24 mo	−1.2	0.1	<0.001†‖
VMMC	0.1	0.1	0.485
Secondary education	−0.5	0.1	<0.001‖
Marital status	0.2	0.1	0.001‖
Premature ejaculation			
12 mo	−0.3	0.1	<0.001†‖
24 mo	−0.7	0.1	<0.001†‖
VMMC	−0.2	0.1	0.001‖
Age 25–29 y	0.3	0.1	0.001‖
Secondary education	0.2	0.1	0.023‖
Employment	0.1	0.1	0.016‖
Marital status	0.3	0.1	<0.001‖
Lack of pleasure during sex			
12 mo	−0.9	0.1	<0.001†‖
24 mo	−1.5	0.1	<0.001†‖
VMMC	0.0	0.1	0.587
Employment	−0.2	0.1	0.010‖
Marital status	0.4	0.1	<0.001‖
Pain during intercourse (dyspareunia)			
12 mo	−0.1	0.1	0.329
24 mo	0.1	0.1	0.711
VMMC	−0.1	0.2	0.623
12 mo × VMMC	−0.9	0.2	<0.001‡‖
24 mo × VMMC	−1.7	0.2	<0.001‡‖
Employment	0.4	0.1	<0.001‖
Condom use at most recent sexual encounter	−0.3	0.1	0.003§‖

VMMC = voluntary medical male circumcision.

∗ Main effects for time (12 and 24 months), group (VMMC), and time-by-group interaction using baseline visit and control group as reference. Time-by-group interaction and other covariates are listed only for a significant α value equal to 0.05. Age categories based on intervals used for recruitment of participants (18–24, 25–29, and 30–35 years) use the youngest age category as reference. All models were adjusted for age, education, employment, and marital status regardless of statistical significance.

† Significant decreases over time in the two study groups.

‡ Significant decrease over time in the VMMC group only.

§ No significant decreases over time in the effect of condom use at the most recent sexual encounter for erectile dysfunction and pain during intercourse. For pain during intercourse, condom use at the most recent sexual encounter was significant only for the VMMC group in stratified models (interaction β = −0.770, *P* < .001).

‖ *P* values less than or equal to .05.

**Table 4:** Changes in sexual satisfaction in circumcised and uncircumcised men over 12 and 24 months of follow-up: results from adjusted random-intercept logistic regression models∗

Outcome and parameters	Estimate (β)	Standard error	Pr > |t|
Satisfaction with sexual intercourse			
12 mo	0.3	0.1	0.022†§
24 mo	0.7	0.1	<0.001†§
VMMC	−0.4	0.1	0.009§
12 mo × VMMC	1.0	0.2	<0.001‡§
24 mo × VMMC	0.7	0.2	<0.001‡§
Secondary education	0.5	0.1	<0.001§
Employment	0.4	0.1	<0.001§
Satisfaction with level of sexual desire			
12 mo	0.1	0.1	0.403
24 mo	0.9	0.2	<0.001†§
VMMC	−0.4	0.2	0.005§
12 mo × VMMC	1.3	0.2	<0.001‡§
24 mo × VMMC	0.9	0.2	<0.001‡§
Age 30–35 y	0.7	0.2	0.002§
Secondary education	0.4	0.1	<0.001§
Employment	0.3	0.1	<0.001§
Satisfaction getting erections			
12 mo	0.4	0.1	0.011†§
24 mo	0.9	0.2	<0.001†§
VMMC	−0.1	0.2	0.416
12 mo × VMMC	1.1	0.2	<0.001‡§
24 mo × VMMC	0.6	0.2	0.012‡§
Secondary education	0.2	0.1	0.039§
Employment	0.4	0.1	<0.001§
Marital status	−0.2	0.1	0.034§
Satisfaction maintaining erections			
12 mo	0.5	0.1	<0.001†§
24 mo	1.3	0.2	<0.001†§
VMMC	−0.0	0.1	0.754
12 mo × VMMC	1.2	0.2	<0.001‡§
24 mo × VMMC	0.5	0.2	0.016‡§
Age 30–35 y	0.6	0.2	0.008§
Employment	0.4	0.1	<0.001§
Marital status	−0.2	0.1	0.015§
Satisfaction with interval between erections			
12 mo	0.5	0.1	0.001†§
24 mo	1.2	0.2	<0.001†§
VMMC	−0.3	0.1	0.034†§
12 mo × VMMC	1.3	0.2	<0.001‡§
24 mo × VMMC	0.8	0.2	0.001‡§
Employment	0.3	0.1	<0.001§
Satisfaction with ease of ejaculation			
12 mo	0.1	0.2	0.395
24 mo	0.9	0.2	<0.001†§
VMMC	−0.3	0.2	0.054†§
12 mo × VMMC	1.5	0.2	<0.001‡§
24 mo × VMMC	0.8	0.2	0.002‡§
Age 25–29 y	0.5	0.2	0.018§
Age 30–35 y	0.7	0.3	0.007§
Employment	0.5	0.1	<0.001§
Marital status	−0.3	0.1	0.002§
Satisfaction with level of pain during intercourse			
12 mo	0.5	0.1	<0.001†§
24 mo	1.5	0.1	<0.001†§
VMMC	−0.2	0.1	0.176
12 mo × VMMC	0.3	0.2	0.031‡§
24 mo × VMMC	0.4	0.2	0.012‡§
Age 25–29 y	−0.4	0.1	0.003§
Secondary education	0.5	0.1	<0.001§
Marital status	−0.2	0.1	0.001§

VMMC = voluntary medical male circumcision.

∗ Main effects for time (12 and 24 months), group (VMMC), and time-by-group interaction using baseline visit and control group as reference. Time-by-group interaction and other covariates are listed only for a significant *P* value less than or equal to .05. Age categories based on intervals used for recruitment of participants (18–24, 25–29, and 30–35 years) use the youngest age category as reference. All models were adjusted for age, education, employment, and marital status regardless of statistical significance.

† Significant increases over time in the two study groups.

‡ Significantly greater increase over time in the VMMC group.

§ *P* values less than or equal to .05.
